# Supplementary material for: High-mobility group box (TOX) antibody a useful tool for the identification of B and T cell subpopulations
Source: PLoS One. 2020 Feb 27;15(2):e0229743. doi: 10.1371/journal.pone.0229743 (PMC7046285; doi:10.1371/journal.pone.0229743)

**Supporting information**

**S1 TEXT**

**Gene expression (GEP)**

GEP data was extracted from previous publications from our group [1, 2]. Batch effect was corrected for Blood and Mod-Pathol datasets using the ComBat algorithm, correction was checked by MDS plot visualization (S1 Fig) [3] and only probes commonly annotated in both datasets were considered in the study. A moderated Student t-test (limma) was used to determine whether the *TOX* gene was differentially expressed in FL (q-value <0.05; absolute fold-change >2.0) (S1 Table) [4]. Statistical analyses and plots were respectively performed and generated using the R environment (R Studio console; RStudio, Boston, MA). Fig 1 and S1 and S2 Figs represents the relative gene expression of the only probe mapping for *TOX* in the two datasets (A_24_P226755).

**Correlation analyses**

Pearson’s correlation was calculated from the Mod-Pathol dataset between each of the two probes mapping for TOX (A_23_P123413 and A_24_P226755) and the rest of the genes. Probes not mapping to *TOX* were previously collapsed to gene symbol by the highest standard deviation. Genes were then ranked by rho correlation coefficient and the top 200 positively (top200-pos) and negatively (top200-neg) correlated genes were selected per each probe.

**Enrichment map**

Enrichment analysis was performed using pre-ranked tool of GSEA software (Gene set enrichment analysis [5] comparing FL vs NMZL in the Blood dataset. Gene-sets were considered statistically enriched when absolute NES >1.2, p <0.05 and FDR <0.25. Enrichment map (S3 Fig) was then generated from the output of the GSEA using EnrichmentMap and AutoAnnotate plugins in Cytoscape as described [6]. Overlap between the corresponding top200-pos or top200-neg per each probe and the statistically enriched gene-sets was computed by Mann-Whitney non-parametric test (p-value <0.001).

**Protein and monoclonal antibody production**

The sequence encoding the TOX protein (NP_055544, residues 1-250) was fused to a C-terminal HIS tag sequence in a T7-based pET21 vector. The protein was produced in *E. coli* BL21 cells. TOX-HIS was purified using a HIS-trap FF column (GE Healthcare) connected to an ÄKTA-prime system (GE Healthcare). The purified fusion protein was concentrated by ultrafiltration.

Two Wistar rats (pathogen free Wistar Han, female, 6 weeks old, 135g weight, Charles River Laboratories, France) were injected intraperitoneally (four times at 14-day intervals) with 100μg of TOX-HIS fusion protein and Complete Freund's adjuvant (Difco). A 150μg last booster of the recombinant TOX-HIS protein was injected intraperitoneally,and splenocytes were fused 3 days later (carbon dioxide was used for euthanasia), as described in [7].

Hybridoma supernatants were screened by ELISA and using HEK-293T cells transfected with pCMV6-MYC-DDK-TOX plasmid. The rat mAb that was raised against TOX (clone NAN448B, IgG1) was cloned by the limiting dilution technique.

Plasmid vectors used to exclude antibody cross reactivity with the TOX2, TOX3 and TOX4 family members are shown in S2 Table [8]. Labelling with the anti-MYC or GFP mAbs confirmed the efficiency of transfections.

**CRISPR/Cas9 Knock-out cells generation**

Viruses were produced by transient plasmid transfection into HEK293T cells using the calcium phosphate method as previously described [9]. Briefly, cells were seeded at 1.1x10^7^ cells/dish in 15cm^2^ dishes the day before transfection. Cells were transfected using calcium phosphate with 3μg pRSV-Rev, 3.75μg pMD.2G (VSV-G), 13μg pMDLg/pRRE, and 35μg of transfer plasmid (pLV-CRISPR). The medium was collected after 48 h, cleared by low-speed centrifugation, and filtered through 0.45-μm pore PVDF filters (Millipore). Viral titers were determined in HEK293T.

Lentiviral supernatants were used to transduce HEK293T and MOLT4 cell lines. Cells were transduced at different multiplicities of infection in medium containing protamine sulphate (25μg/ml final concentration). Cells were incubated at 37^o^C for 6h and the viral supernatant was then replaced with fresh medium and selection with puromycin was started at 72h post-transduction. After 10 days, cells were sorted in a 96-well plate in order to establish single-cell clones. Proteins were isolated from each colony and western blot was performed to identify for KO cells. In those that lack expression of TOX the target region was analyzed by sanger sequencing.

**Western blotting (total protein extraction and blot visualization)**

WB was performed with total cell extracts of human lymphoma and HEK293T cell lines. The total lysates of each cell line were denatured by heating in Laemmli sample buffer, resolved on a 10% sodium dodecyl sulphate-polyacrylamide gel (SDS-PAGE) and transferred onto nitrocellulose membranes for 2h. The blots were visualized using the ECL detection system (Amersham Biosciences, Buckinghamshire, UK) in accordance with the supplier’s instructions.

**Double immunenzymatic staining**

For paraffin embedded tissues an automated dewaxing and rehydration step followed by heat-induced (98°C for 20 min) and enzyme-induced antigen retrieval (10 to 15 min, Bond Enzyme Pre-treatment Kit, Leica, Germany) was performed. Heat-induced antigen retrieval was performed using Tris/EDTA buffer, pH9 ready-to-use solution (Leica, Germany). The slides were subsequently incubated with 3% hydrogen peroxide (5 min), primary antibodies (S3 Table) (30 min), a post-primary blocking reagent (to prevent nonspecific polymer binding) (8 min), horseradish peroxidase (HRP)-labeled rabbit anti-rat, goat anti-rabbit and rabbit anti-mouse Ig polymer (8 min), and diaminobenzidine substrate (10 min). All reagents were components of the Bond Polymer Refine detection system (Leica, Germany). A second immuno-alkaline phosphatase (AP) procedure was then performed, omitting the dewaxing, rehydration, and epitope retrieval steps. The primary antibodies (S3 Table) were applied for 40 min followed by incubation with post-primary AP (alkaline phosphatase) blocking reagent (20 min) and AP-labeled specific secondary antibodies (30 min), both of which are components of the Bond Polymer AP Red detection system (Leica, Germany). The AP reaction was developed with either the Fast Red substrate included in the Bond Polymer AP Red detection system. Hematoxylin counterstaining was performed.

**Immunofluorescence**

After antigen retrieval (Tris-EDTA buffer), the slides were incubated for one hour at room temperature in a humid chamber with primary antibodies (anti-TOX NAN448A, anti-BCL6 and anti-Ki67). Slides were then washed in PBS 0.5%-Tween20 (Sigma, Germany) 3 times for 5 min each. The slides were incubated for 1 hour with fluorochrome-conjugated antibodies (Alexa Fluor 594 and Alexa Fluor 488, dilution 1:200) against the different species, diluted in PBS (Molecular Probes, Leiden Netherlands) in a humid chamber in the dark. Subsequently, slides were washed in PBS 0.5% Tween20 (Sigma, Germany) 3 times for 5 min each. Following washing, antifading medium with dapi (Qbiogene, Illkirch, FR) was added.

Slides were examined on a Nikon E800 Eclipse fluorescence microscope (Nikon, Kingston-upon-Thames, UK) equipped for epifluorescence. Fluorescence images were captured with an Axiocam charge-coupled device (CCD) camera (Zeiss, Jena, Germany) and Axiovision software (Imaging Associates, Bicester, UK), and adjusted using Photoshop software (Adobe, San Jose, CA, USA).

**TOX expression in non-lymphoid tissues**

TOX expression was also analyzed in human normal tissues (intestine n=6, lung n=5, kidney n=5, ovary n=4, pancreas n=5, testis n=5, brain n=6, prostate n=4, thyroid n=2, breast n=3, bladder n=2, placenta n=2, fallopian tube n=2) (S4 Fig). TOX protein was detected in undifferentiated crypt cells and in myenteric plexus of the intestine while in lung expression was restricted to isolated pneumocytes. TOX was also expressed in the distal convoluted tubule of the kidney and in the functional stroma in the ovary. Endocrine cells of the pancreas and Sertoli cells of the testicle were also strongly TOX positives. No TOX expression was found in the other tissues analyzed, with the exception of a weak staining in the brain cortex.

**Tox expression in mouse tissue**

Tissue samples from male C57BL/6 mice (6 months old and 15.5 days post coitum embryo, Harlan Laboratories, USA) were fixed in 10% neutral buffered formalin (4% formaldehyde in solution), paraffin-embedded and cut at 3μm, mounted on superfrost®plus slides (Thermo-Fisher, Germany) and dried overnight. IHC reactions were performed in an automated immunostaining platform (Autostainer Link 48, DAKO, Denmark).

Antigen retrieval was first performed with high pH buffer and endogenous peroxidase was blocked (peroxide hydrogen at 3%). Then, slides were incubated with rat monoclonal anti-TOX (NAN448B, neat supernatant). Slides were then incubated with the secondary antibodies rabbit anti rat (Vector, USA) and visualization systems (Novolink Polymer, Bond, Leica, Germany) conjugated with HRP. IHC reaction was developed using 3, 30-diaminobenzidine tetrahydrochloride (DAB) (DAKO, Denmark) and nuclei were counterstained with Carazzi’s hematoxylin. Finally, the slides were dehydrated, cleared and mounted with a permanent mounting medium for microscopic evaluation. Positive control sections known to be primary antibody positive were included for each staining run. Whole slides were acquired with a slide scanner (AxioScan Z1, Zeiss, Germany).

As expected, Tox expression in mouse adult tissues was very similar to that observed in human tissues. Tox expression was found in lymph node (T cells in the interfollicular areas and GC B and T cells), thymus (cortex lymphoblast and some B and T cells in the medulla), spleen (germinal center B and T cells and in some cells in the red pulp), brain (neurons of the cerebral cortex), testicle (Sertoli cells), pancreas (endocrine cells) and a weak staining in kidney (distal convoluted tubule). In mouse embryo Tox-positive cells were found in the foetal liver suggesting Tox involvement in B-cell development (S5 Fig).

**Supplemental references**

1.Arribas AJ, Gomez-Abad C, Sanchez-Beato M, Martinez N, Dilisio L, Casado F, et al. Splenic marginal zone lymphoma: comprehensive analysis of gene expression and miRNA profiling. Mod Pathol. 2013;26:889-901. PMID: 23429603.

2.Arribas AJ, Campos-Martin Y, Gomez-Abad C, Algara P, Sanchez-Beato M, Rodriguez-Pinilla MS, et al. Nodal marginal zone lymphoma: gene expression and miRNA profiling identify diagnostic markers and potential therapeutic targets. Blood. 2012;119:e9-e21. PMID: 22110251.

3.Leek JT, Storey JD. Capturing heterogeneity in gene expression studies by surrogate variable analysis. PLoS Genet. 2007;3:1724-35. PMID: 17907809.

4.Gentleman RC, Carey VJ, Bates DM, Bolstad B, Dettling M, Dudoit S, et al. Bioconductor: open software development for computational biology and bioinformatics. Genome Biol. 2004;5:R80. PMID: 15461798.

5.Subramanian A, Tamayo P, Mootha VK, Mukherjee S, Ebert BL, Gillette MA, et al. Gene set enrichment analysis: a knowledge-based approach for interpreting genome-wide expression profiles. Proc Natl Acad Sci U S A. 2005;102:15545-50. PMID: 16199517.

6.Reimand J, Isserlin R, Voisin V, Kucera M, Tannus-Lopes C, Rostamianfar A, et al. Pathway enrichment analysis and visualization of omics data using g:Profiler, GSEA, Cytoscape and EnrichmentMap. Nat Protoc. 2019;14:482-517. PMID: 30664679

7.Roncador G, Brown PJ, Maestre L, Hue S, Martinez-Torrecuadrada JL, Ling KL, et al. Analysis of FOXP3 protein expression in human CD4+CD25+ regulatory T cells at the single-cell level. Eur J Immunol. 2005;35:1681-91. PMID: 15902688.

8.Seiler CY, Park JG, Sharma A, Hunter P, Surapaneni P, Sedillo C, et al. DNASU plasmid and PSI:Biology-Materials repositories: resources to accelerate biological research. Nucleic Acids Res. 2014;42:D1253-60. PMID: 24225319.

9.Torres-Ruiz R, Martinez-Lage M, Martin MC, Garcia A, Bueno C, Castano J, et al. Efficient Recreation of t(11;22) EWSR1-FLI1(+) in Human Stem Cells Using CRISPR/Cas9. Stem Cell Reports. 2017;8:1408-20. PMID: 28494941.

**S1 Figure.** Multi-dimensional plot for the top 5000 most variable probes, before (not corrected, left panel) and after (Combat corrected, right panel) the batch correction for the Blood (black dots) and Mod-Pathol (grey dots) datasets. Batch effect was corrected using the ComBat algorithm of the “sva” R package.

**S1 Table**. Differential expression of the *TOX* gene in FL by moderated Student t-test (limma). FL was independently compared to NMZL, MALT marginal zone lymphoma, CLL, MCL, SMZL and reactive lymphoid tissue: lymph node and spleen. The column symbol_probe represents the gene symbol (*TOX*) and the probe ID (A_24_P226755) respectively; p-value for nominal p-value; q-value represents the adjusted p-value for multiple testing (Bonferroni); FL avg for the mean of FL cases; Other classes avg for the mean of the corresponding class, specified in the Comparison column; log2 FC for log2 scaled fold-change between FL mean and the corresponding class mean; FC for fold-change between FL mean and the corresponding class mean.

| Comparison | symbol_probe | p-value | q-value | FL avg | Other classes avg | log2 FC | FC |
| --- | --- | --- | --- | --- | --- | --- | --- |
| FL vs NMZL | TOX_A24P226755 | 8.090E-08 | 4.600E-07 | 1.827 | 0.177 | 1.651 | 3.140 |
| FL vs MALT |  | 4.800E-06 | 2.120E-05 |  | 0.770 | 1.057 | 2.081 |
| FL vs CLL |  | 2.164E-02 | 4.194E-02 |  | -0.545 | 2.373 | 5.178 |
| FL vs MCL |  | 2.048E-04 | 1.450E-03 |  | -1.623 | 3.450 | 10.929 |
| FL vs SMZL |  | 1.462E-07 | 8.685E-07 |  | -0.672 | 2.500 | 5.656 |
| FL vs reac. Lymph Node |  | 3.650E-11 | 4.280E-10 |  | 0.509 | 1.319 | 2.495 |
| FL vs reac. Spleen |  | 2.650E-10 | 2.370E-09 |  | -1.292 | 3.120 | 8.693 |

**S2 Figure**. Barplot on the expression data from each individual case. Bars represent the relative gene expression of the only probe mapping for *TOX* in both datasets (A_24_P226755). FL: follicular lymphoma (blue); MALT: mucosa-associated lymphoid tissue (MALT) marginal zone lymphoma (yellow); lymp node (grey); NMZL: nodal marginal zone lymphoma (red); SMZL: splenic marginal zone lymphoma (green); CLL: chronic lymphocytic leukemia (purple); MCL: mantle cell lymphoma (magenta); and reactive lymphoid tissues: lymph node (grey) and spleen (light blue).

**S3 Figure**. Enrichment map from gene expression profiling of FL. Node size represents the size (number of genes) of the gene-sets and signatures. Red or blue nodes correspond to the statistically enriched gene-sets: blue for FL-enrichment and red for NMZL-enrichment. The previously identified NMZL signatures were included in the enrichment map: overexpressed genes in NMZL when compared either to FL (UP NMZL vs FL) or reactive lymph node (UP NMZL vs RLN). Grey nodes represents the top-200 positively or top-200 negatively correlated genes (top200s) per each *TOX* probe: “pos- or neg-corr TOX A23 probe” for A_23_P123413, and “pos- or neg-corr TOX A23 probe” for A_24_P226755 respectively. Overlap between gene-sets, signatures and top200s *TOX*-correlated genes was computed by Mann-Whitney non-parametric test. Only significant overlap is shown in the map (p-value <0.001). Light blue edges represents overlap between gene-sets and orange edges represents overlap between gene-sets and signatures, either NMZL signatures or top200s *TOX*-correlated genes. Abbreviations are detailed in the corresponding image legend.

**S2 Table**. Plasmid vectors used in the study. DNASU Plasmid source information.

| **Molecule** | **Protein sequence** | **Plasmid** | **Tag** | **Source** |
| --- | --- | --- | --- | --- |
| TOX (isoform1) | Full length | pCMV6 | MYC-DDK | Origene |
| TOX2 (isoform2) | Full length | pCDNA3 | GFP | DNASU-PDONR221 |
| TOX3 (isoform1) | Full length | pCDNA3 | GFP | DNASU-PENTR223.1 |
| TOX4 (isoform1) | Full length | pCDNA3 | GFP | DNASU-PDONR221 |

**S3 Table**. Antibodies used in the study.

| **Molecule** | **Antibody type** | **Clone name** | **Dilution** | **Source** |
| --- | --- | --- | --- | --- |
| TOX | Rat monoclonal | NAN448B | 1:100 supernatant | CNIO |
| MYC | Mouse monoclonal | 9E10 | 1:100 | Abcam |
| GFP | Rat monoclonal | LAS325A | Neat supernatant | CNIO |
| Vinculin | Mouse monoclonal | Hvin-1 | 1:10000 | Sigma |
| BCL6 | Mouse monoclonal | IG191 | 1:350 | CNIO |
| CD10 | Mouse monoclonal | 56C6 | Prediluted | DAKO |
| CD30 | Mouse monoclonal | CON6D | 1:10 supernatant | CNIO |
| CD68 | Mouse monoclonal | PGM1 | 1:1000 | DAKO |
| CD138 | Mouse monoclonal | CRUZ180A | 1:100 | CNIO |
| IgD | Rabbit polyclonal | Polyclonal | Prediluted | DAKO |
| PD1 | Mouse monoclonal | NAT105 | 1:4 supernatant | CNIO |
| CD3 | Rabbit polyclonal | Polyclonal | Prediluted | DAKO |
| CD4 | Mouse monoclonal | 4B12 | Prediluted | DAKO |
| CD8 | Rat monoclonal | NOR132H | 1:5 supernatant | CNIO |
| KI67 | Mouse monoclonal | MIB-1 | Prediluted | DAKO |

**S4 Fig.** TOX expression in normal human tissues.

**
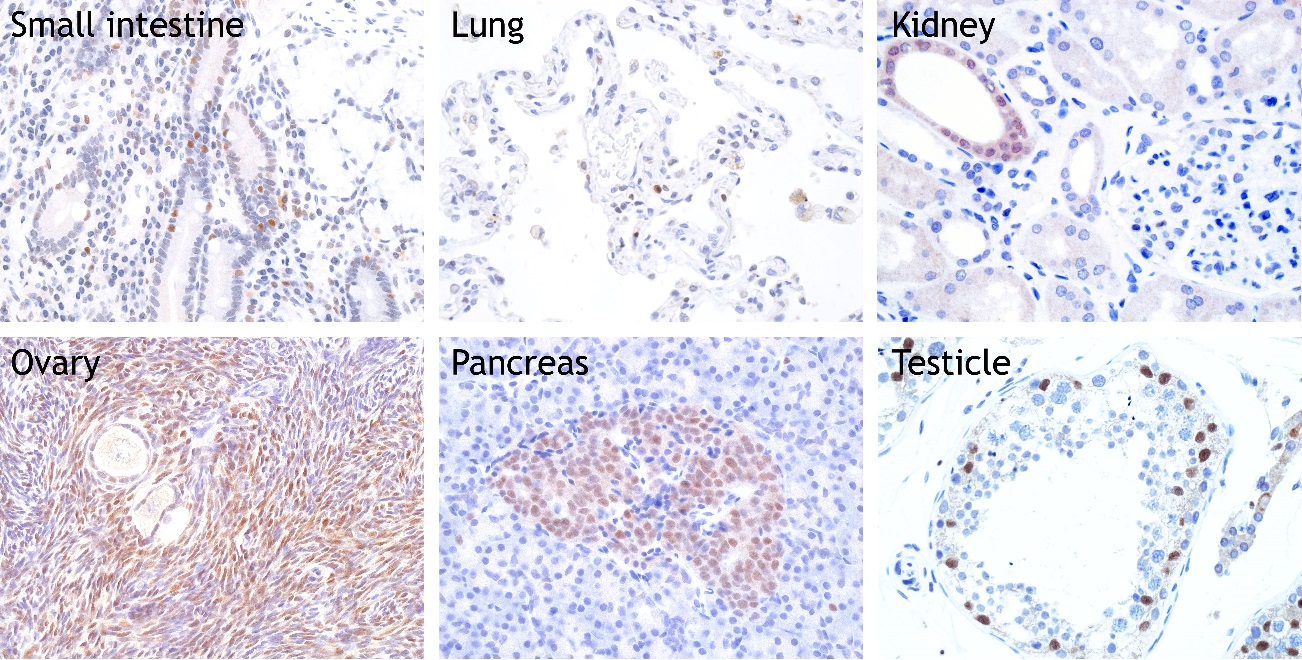
**

**S5 Fig.** Immunostaining of Tox protein in normal adult and fetal mouse tissues.


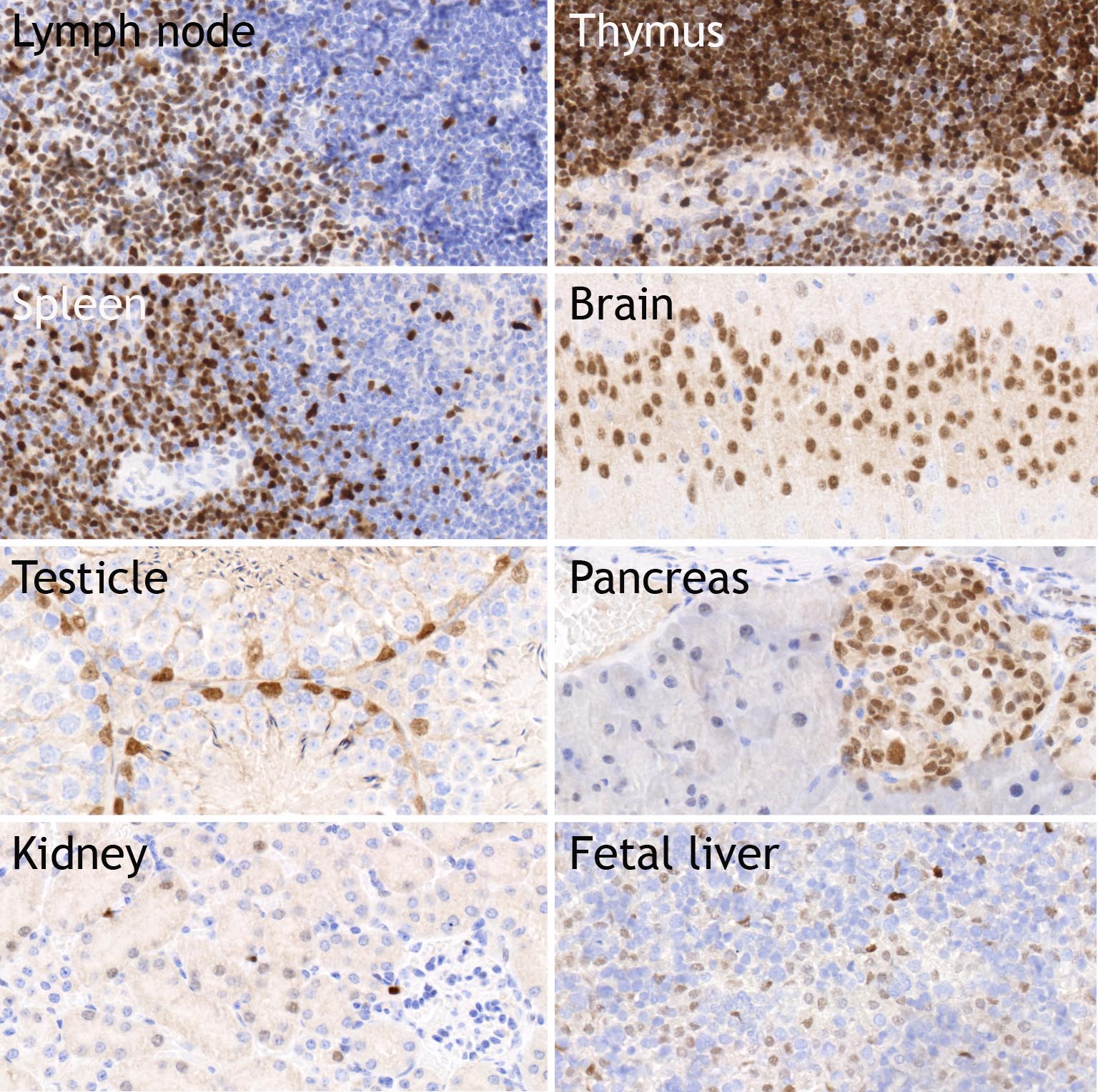


**S6 Figure.** Raw Western Blotting images


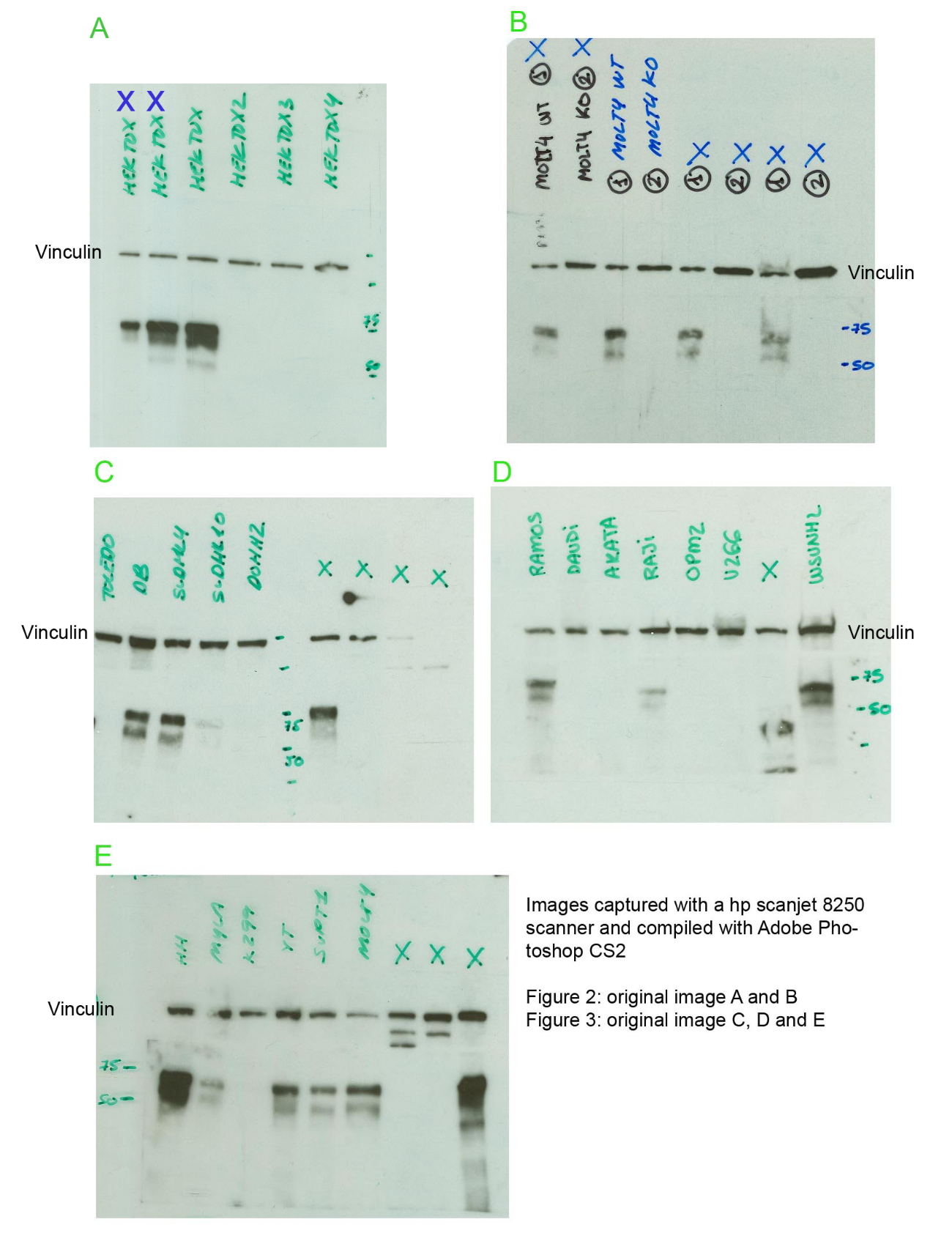


**S7 Figure.** Cell line authentication report.


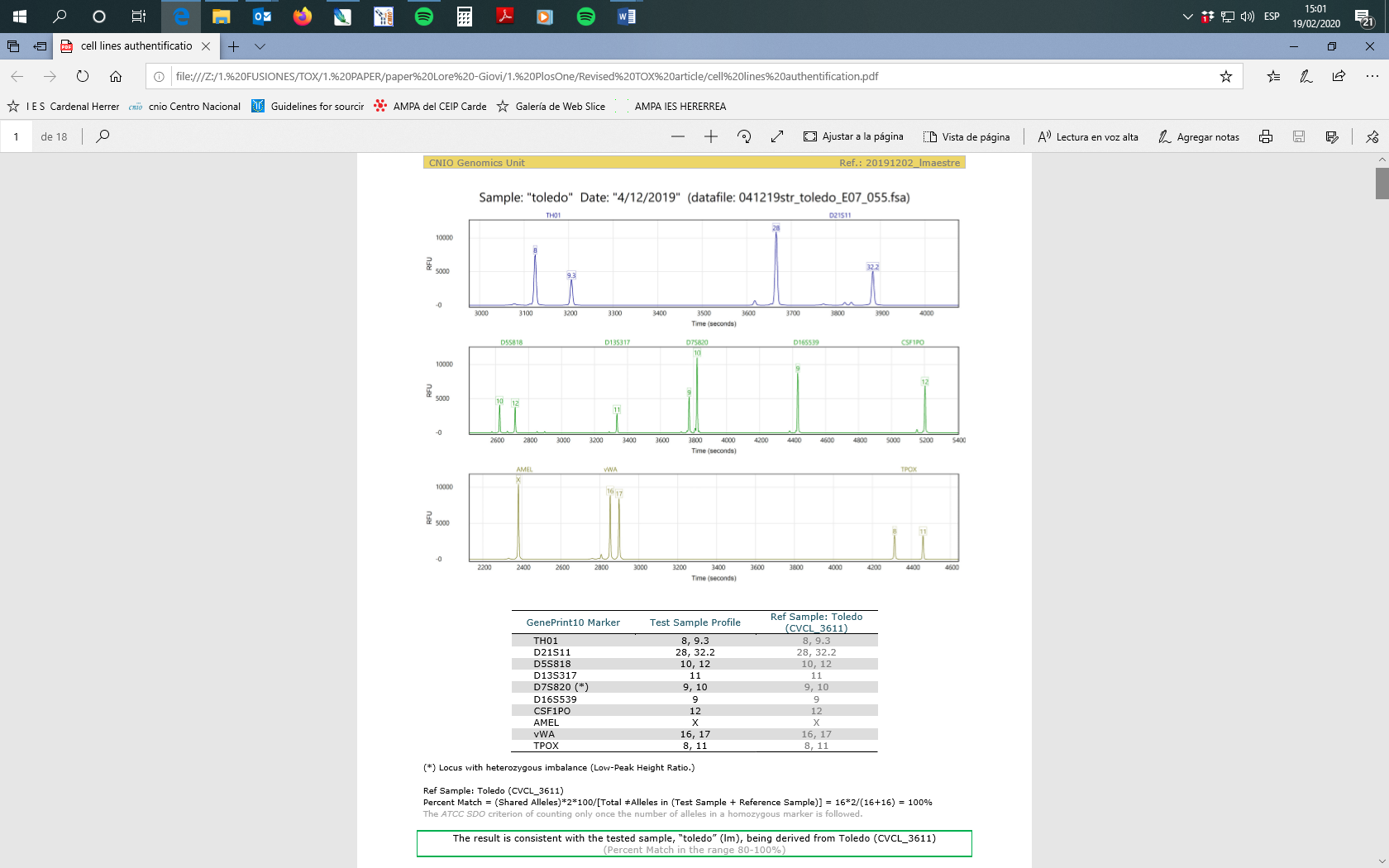


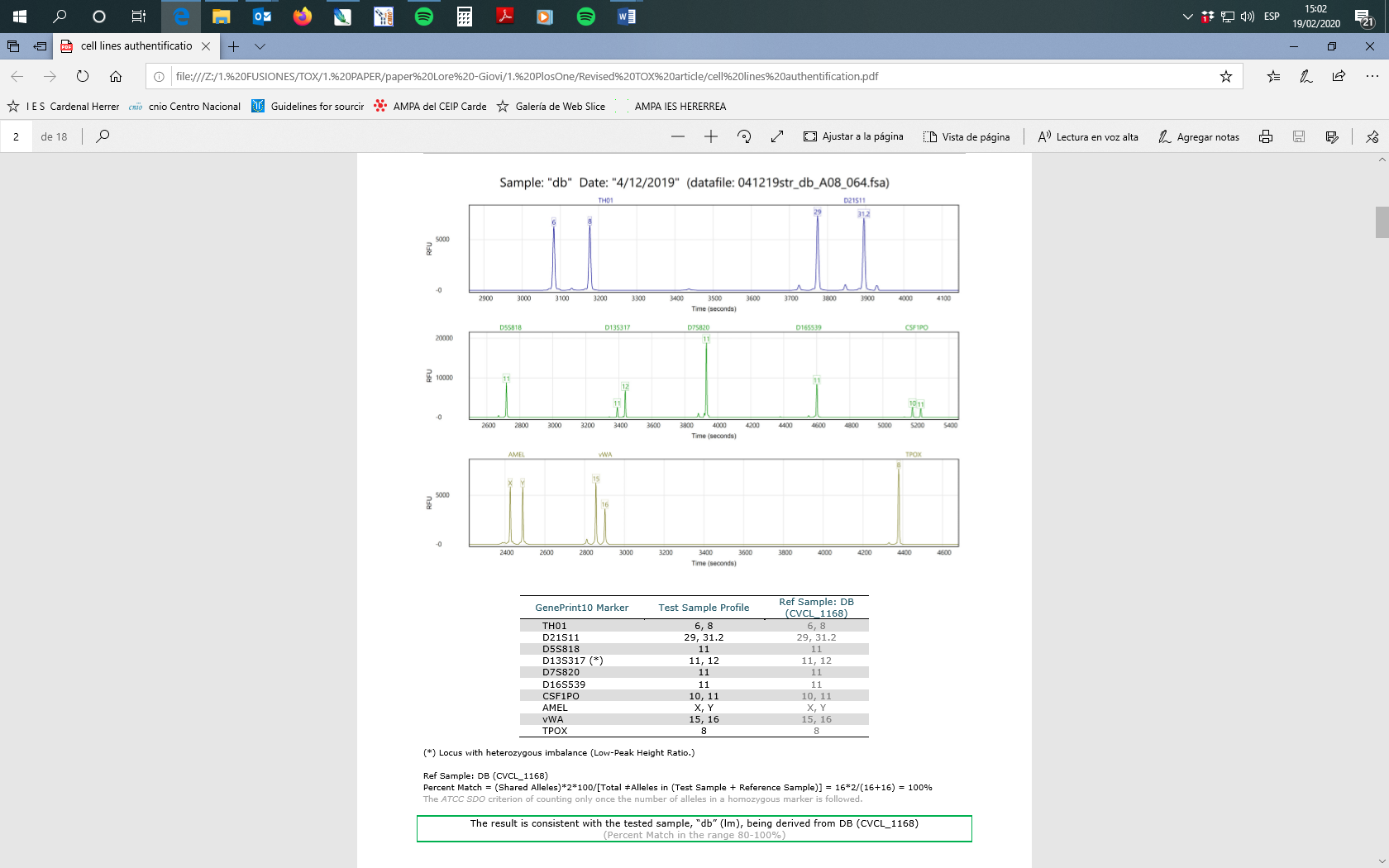


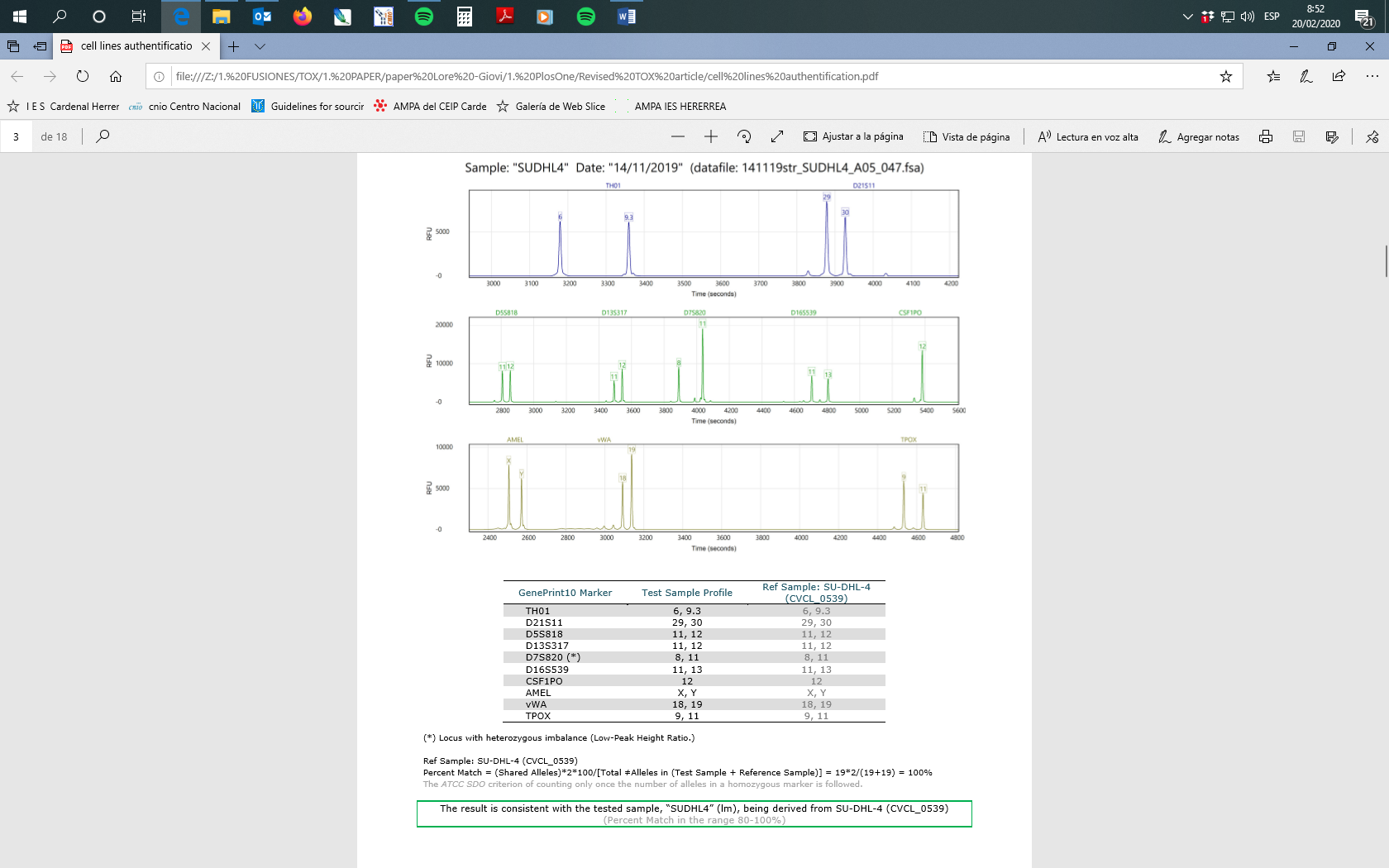


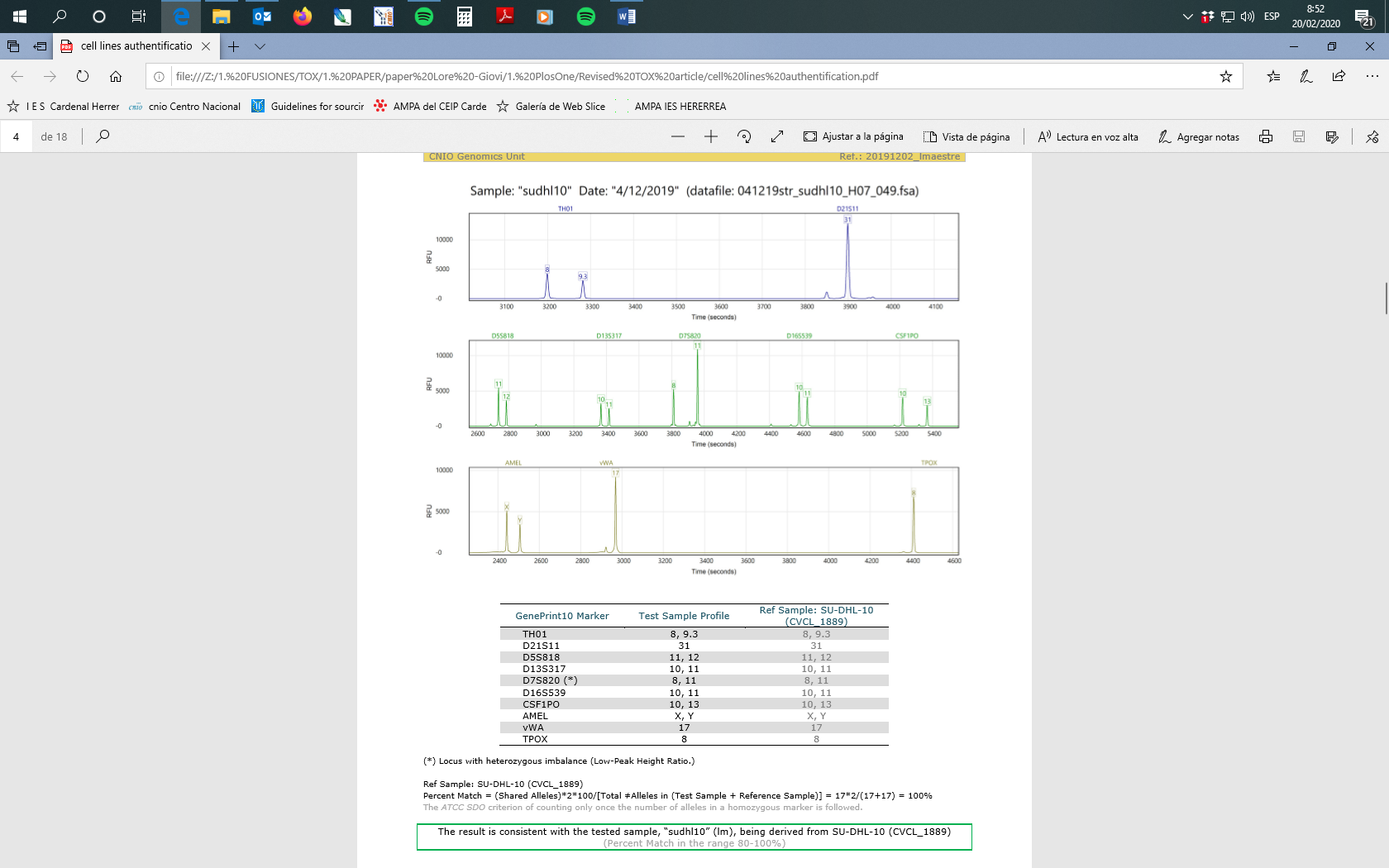


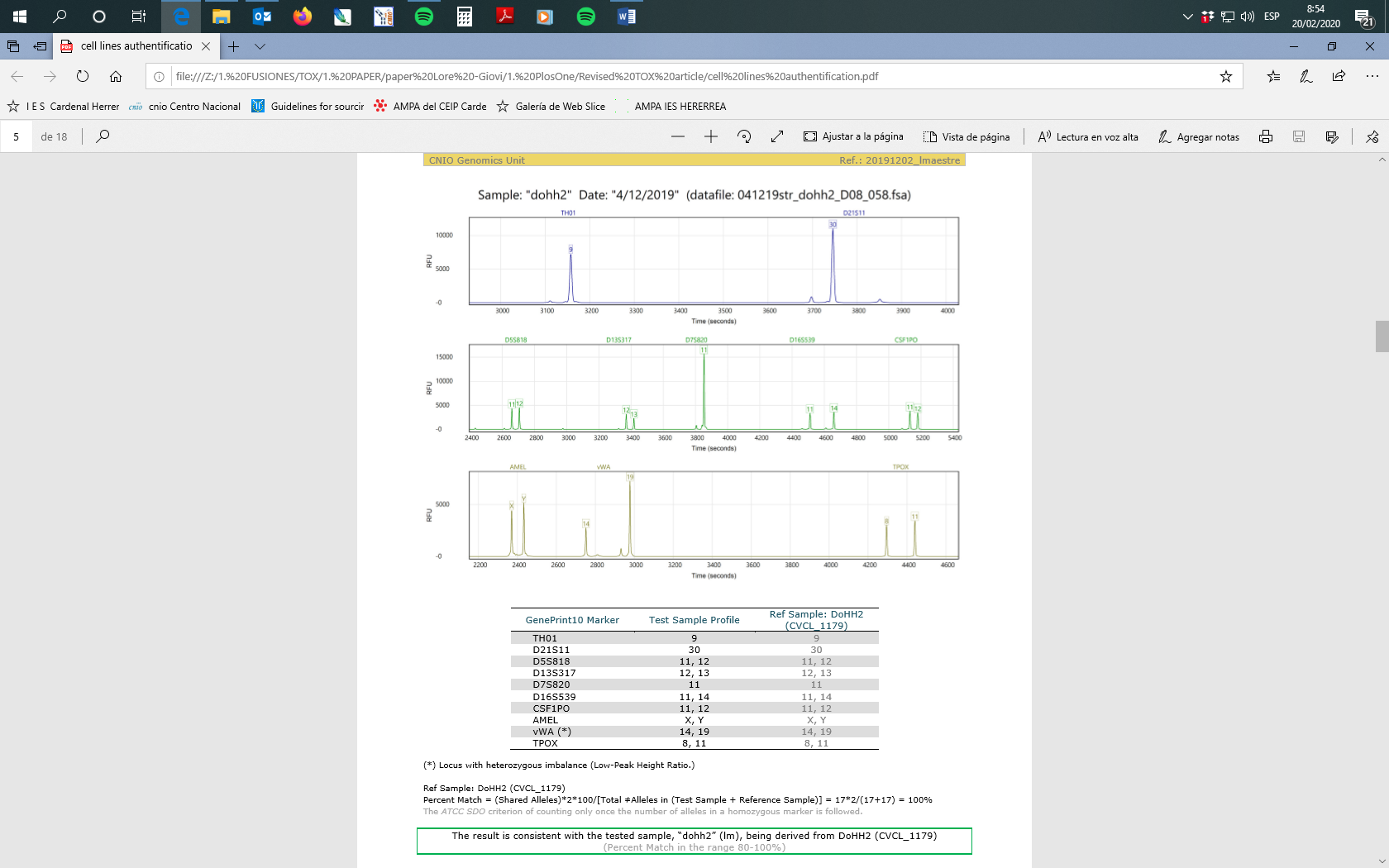


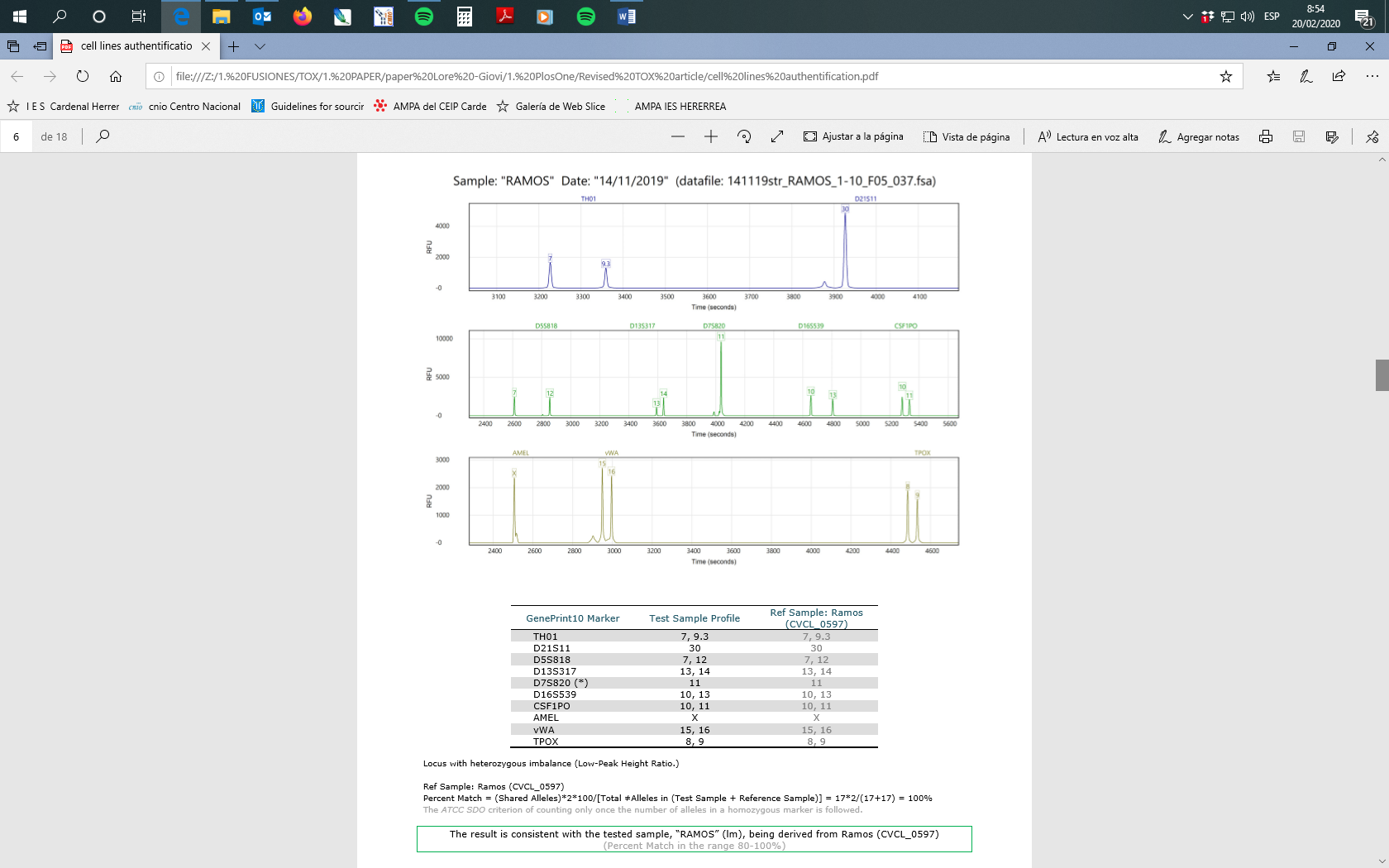


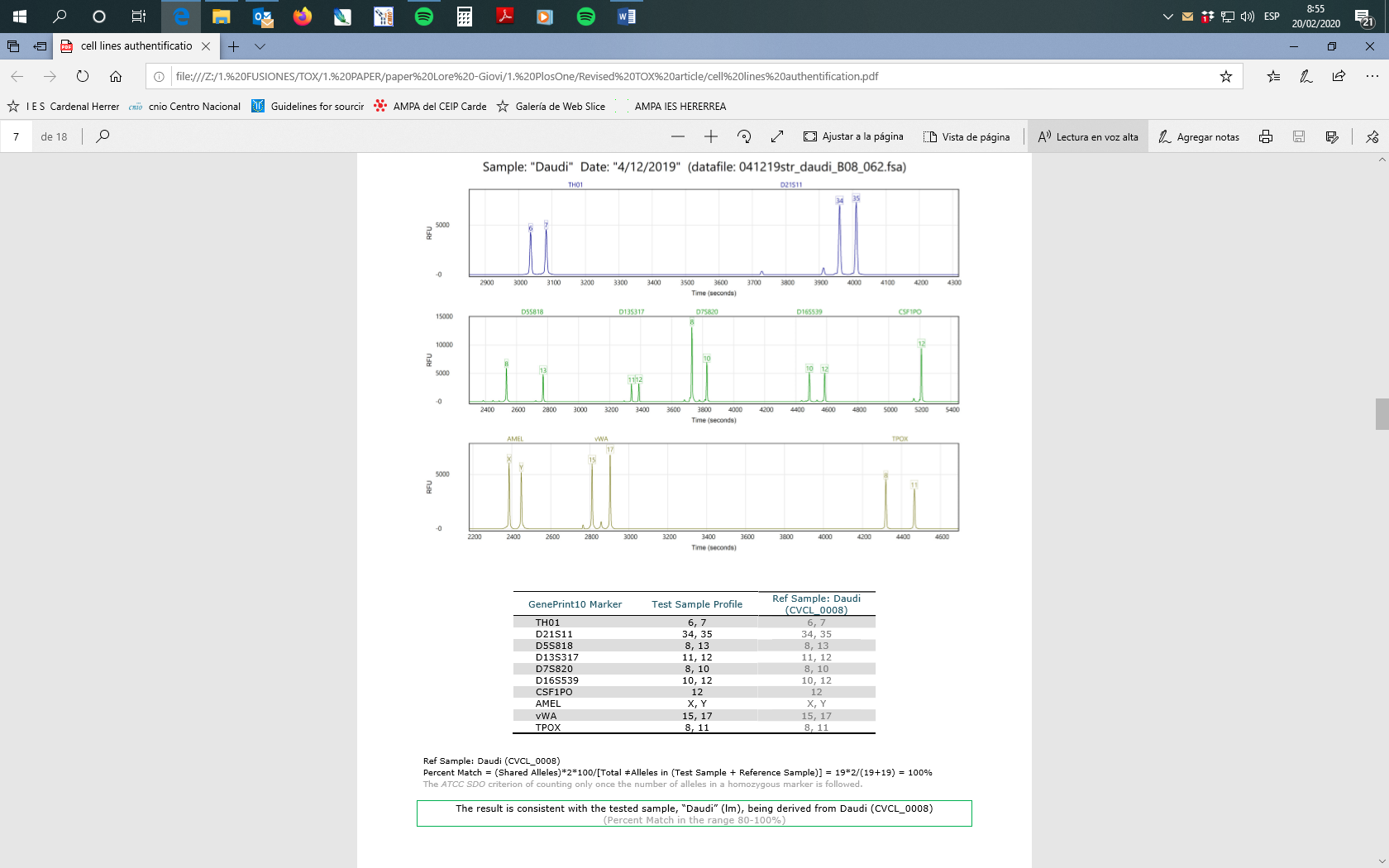


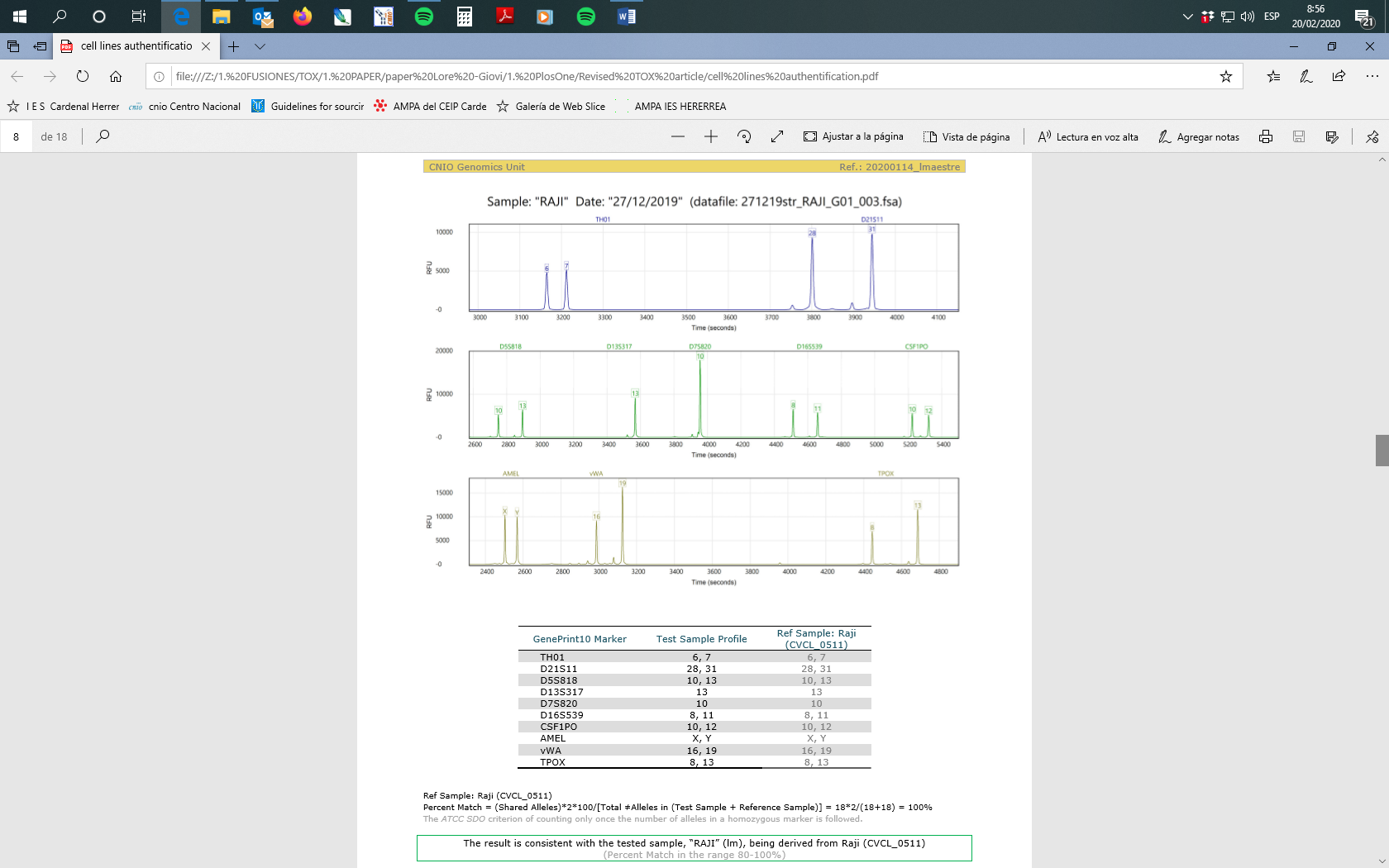


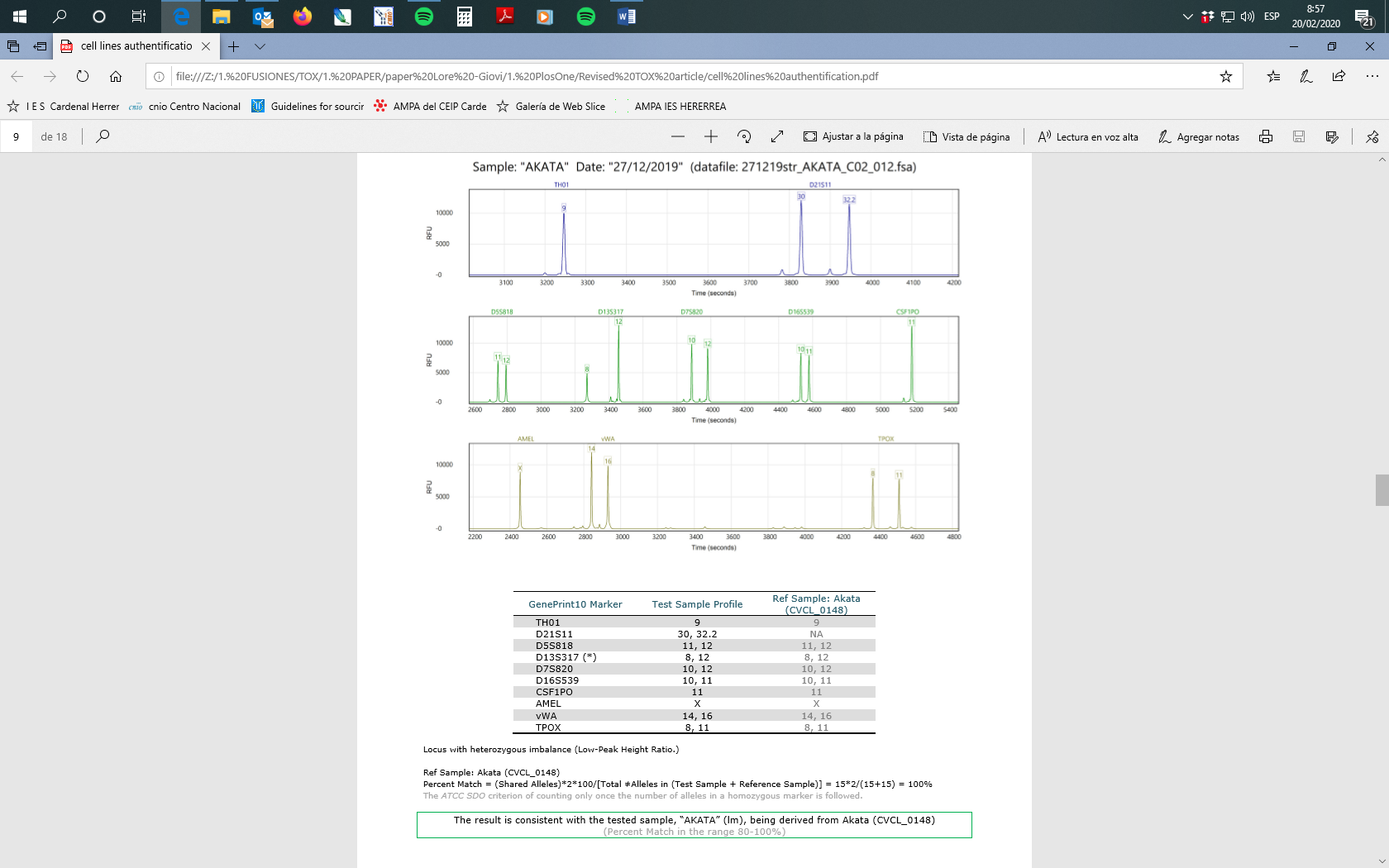


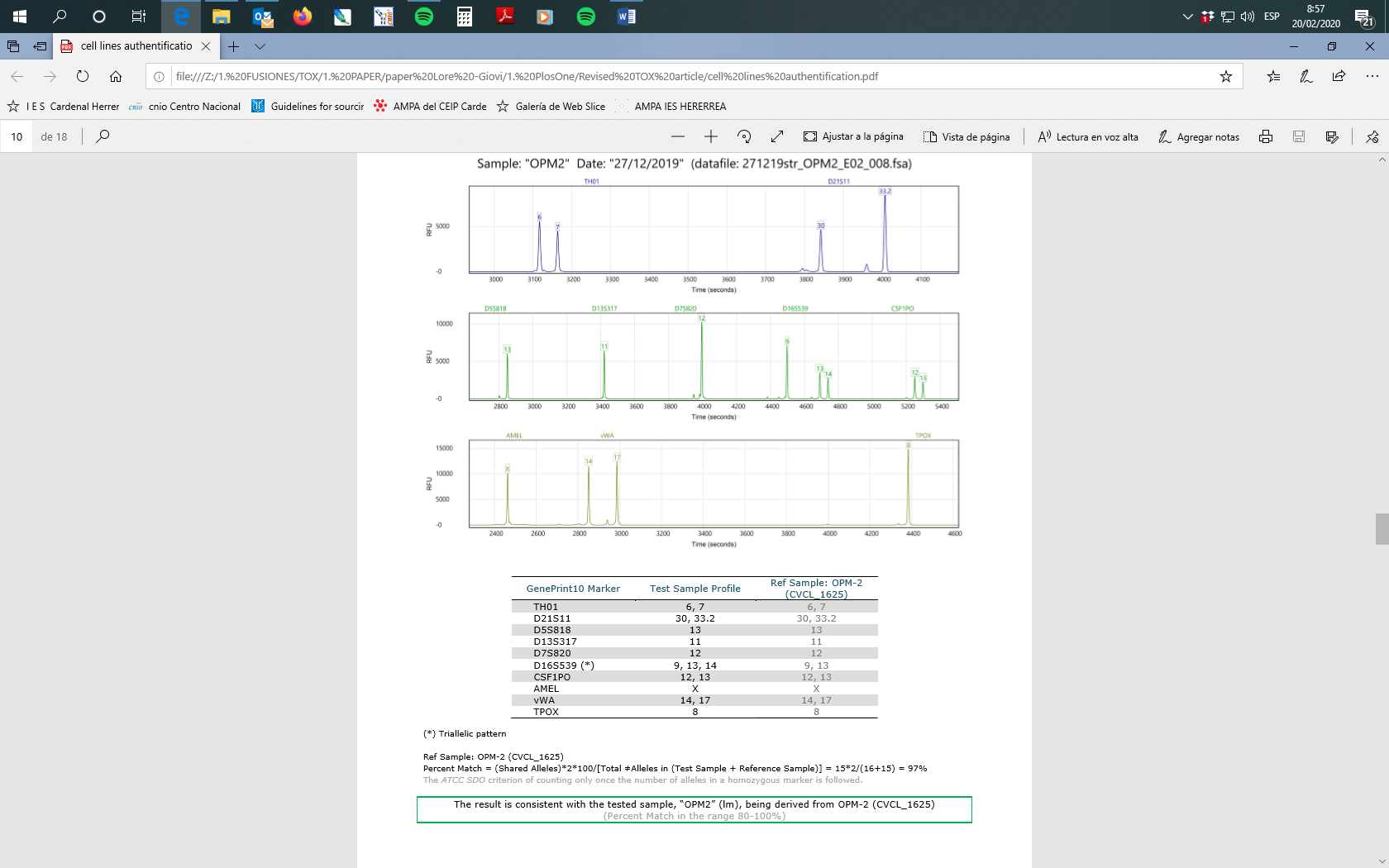


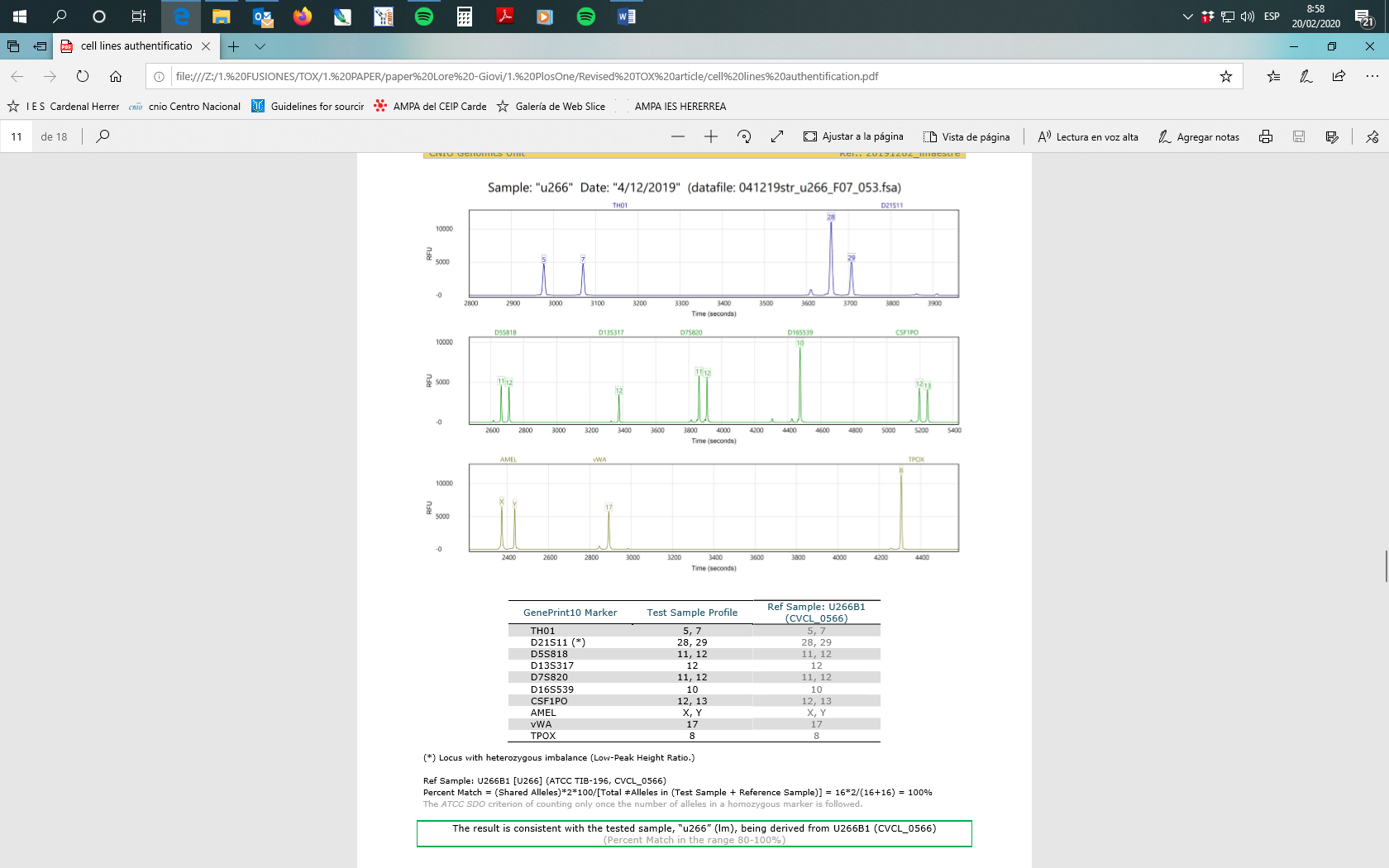


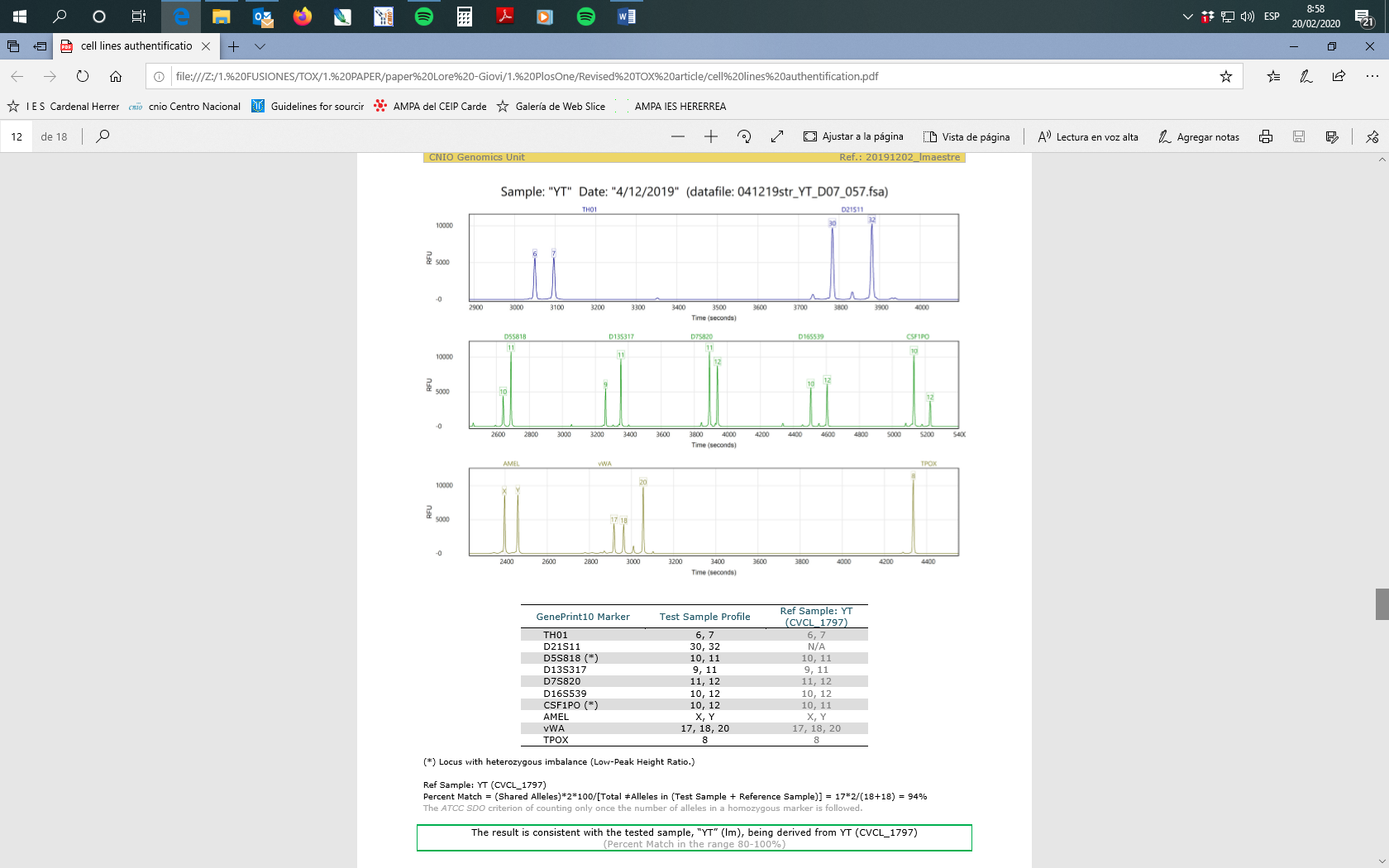


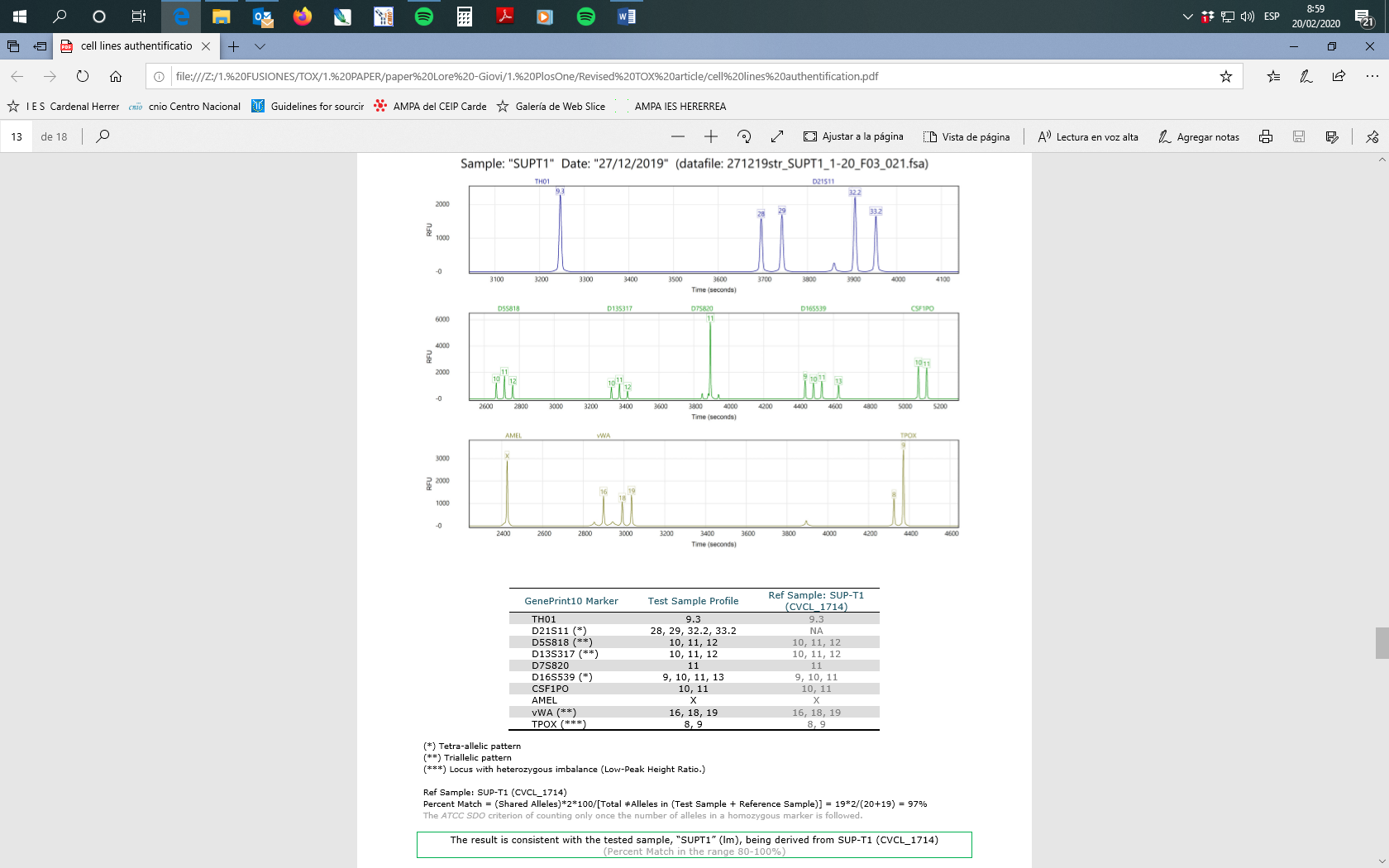


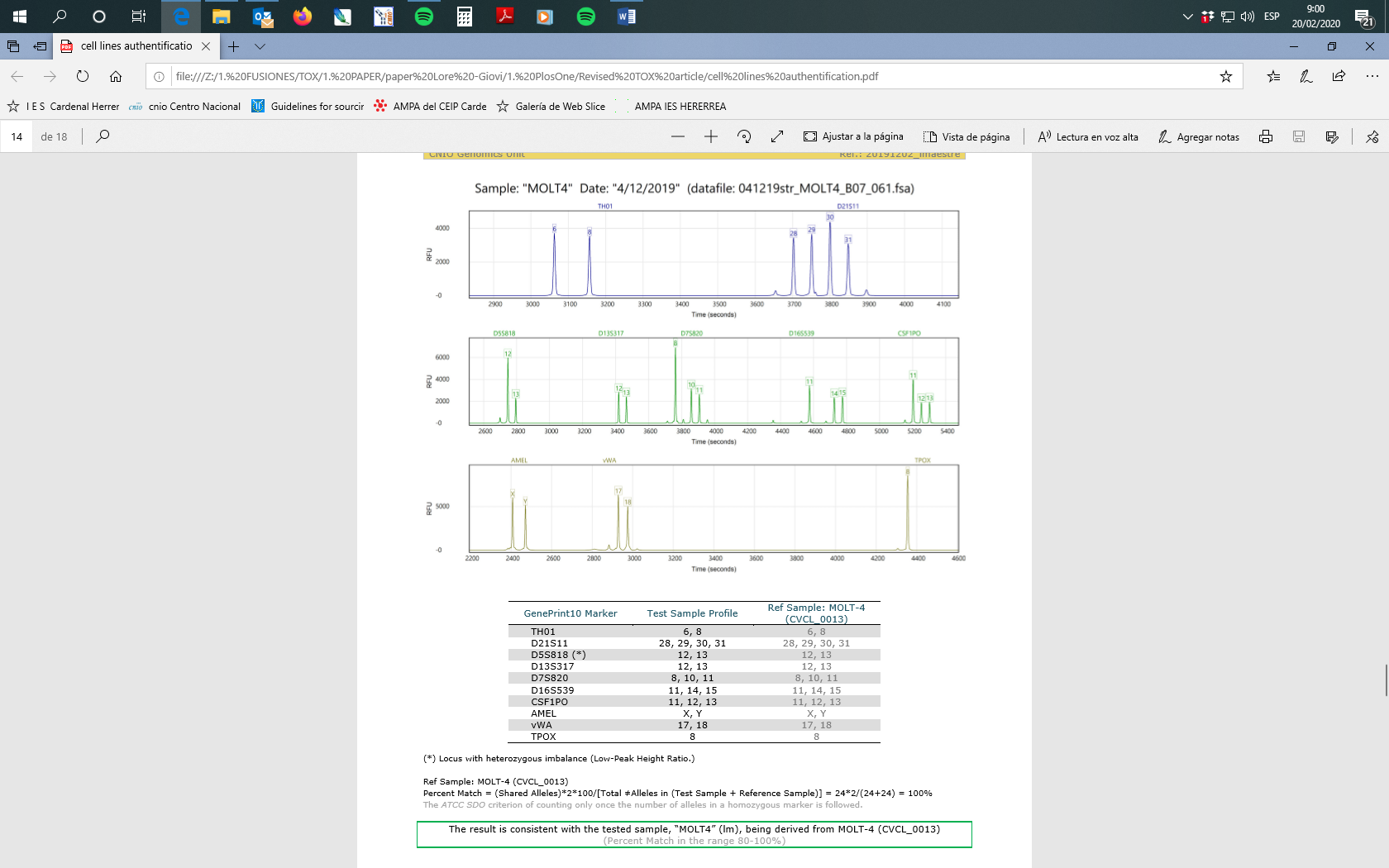


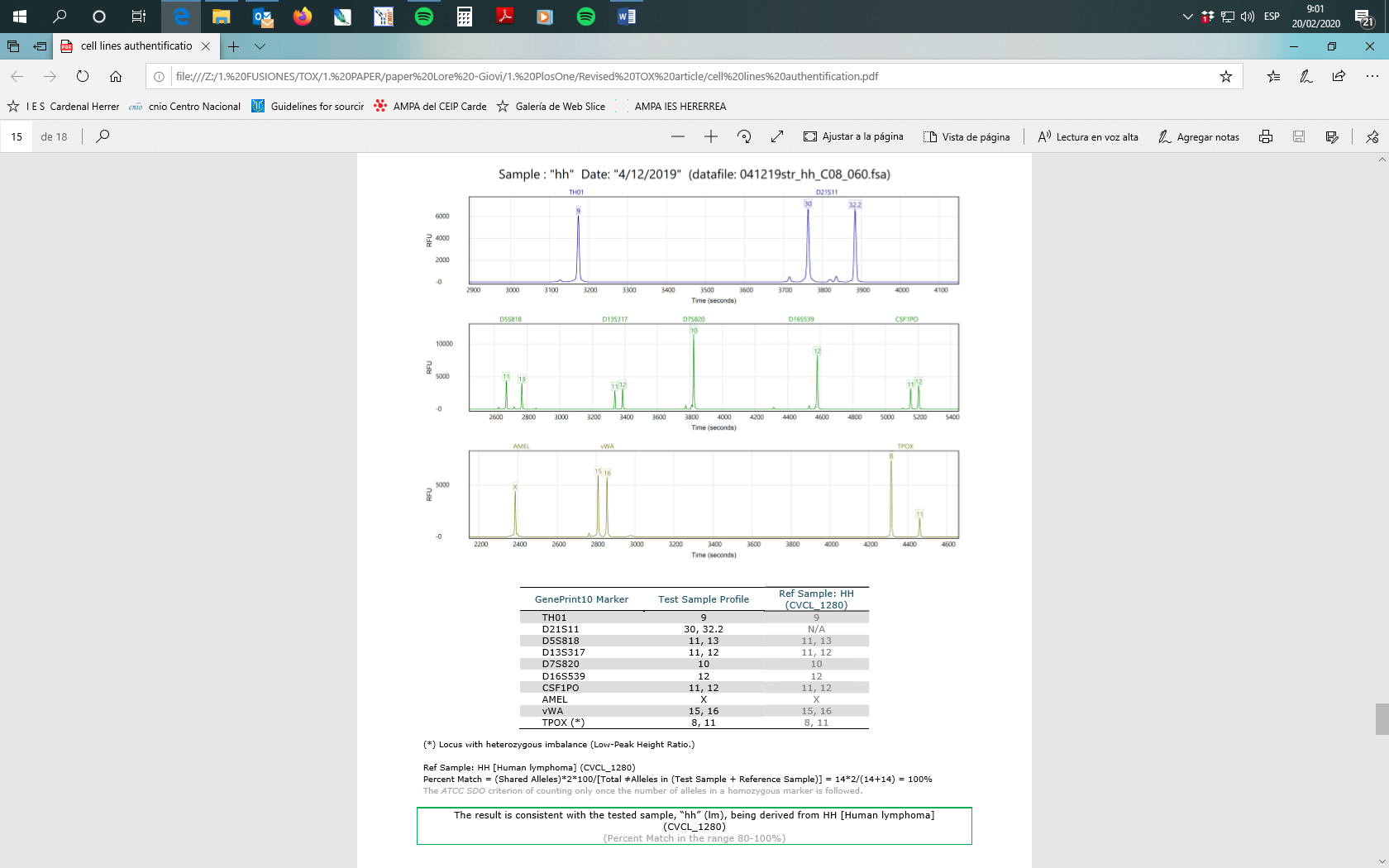


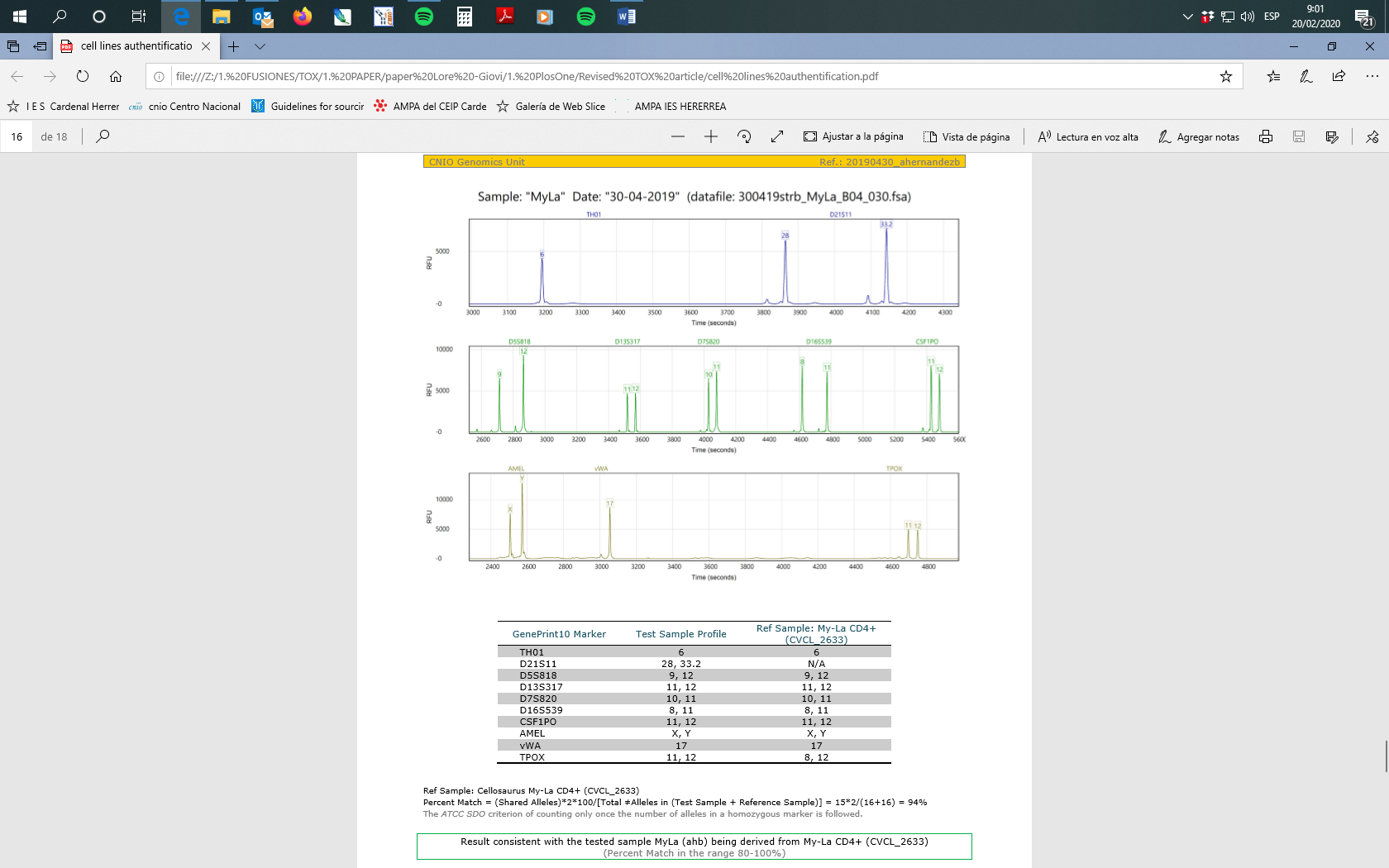


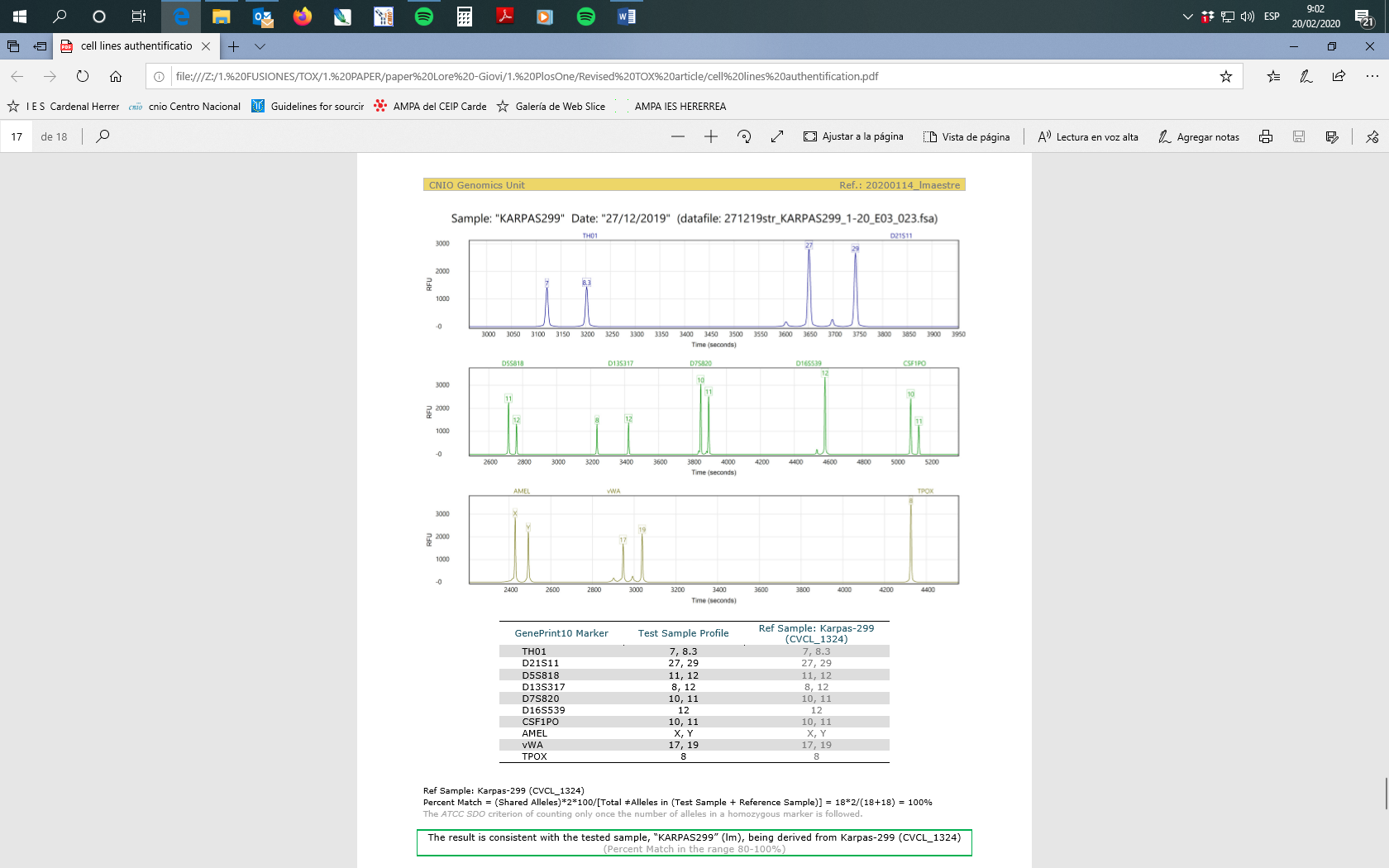


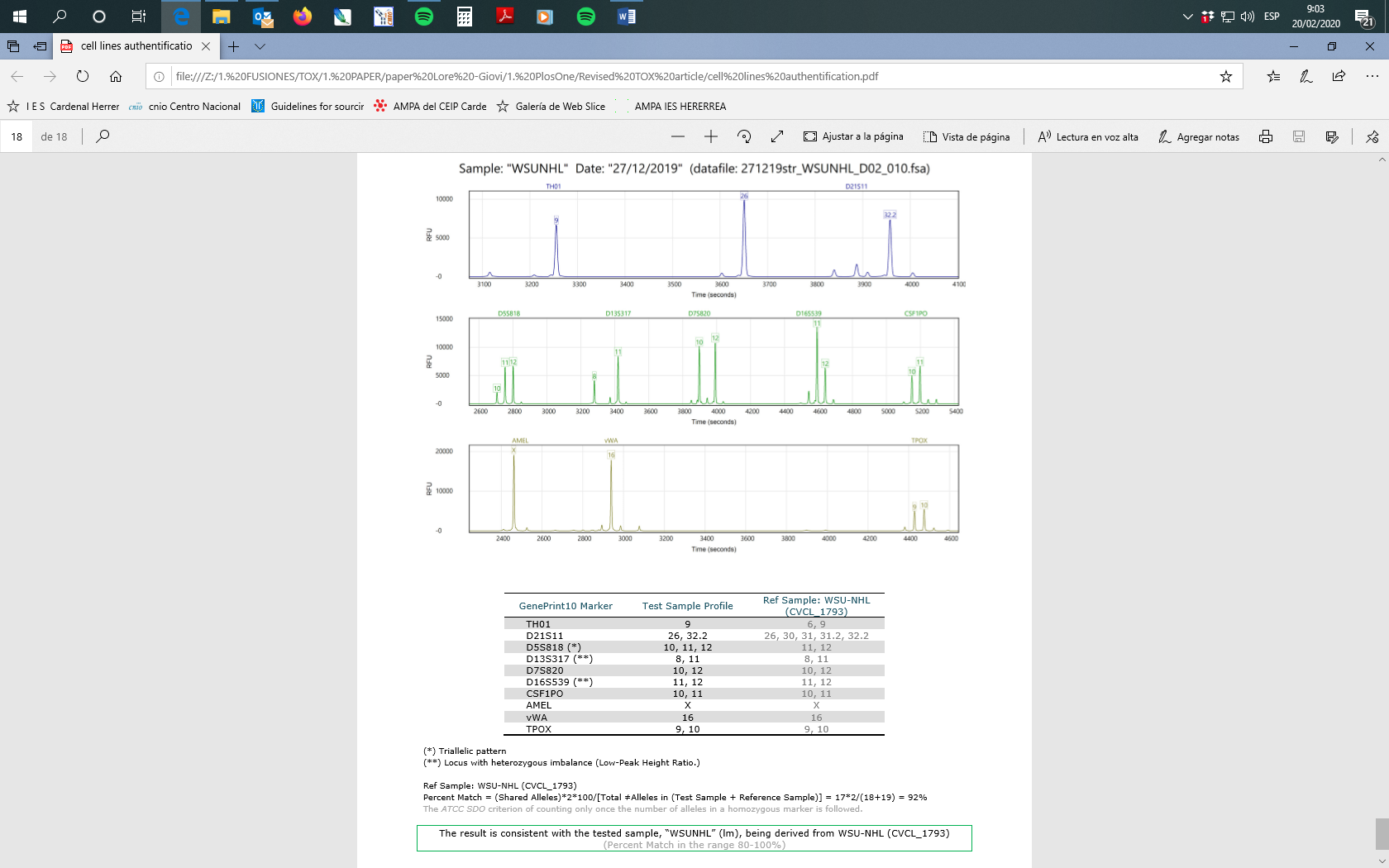

Supplement: S1 Text — (DOC) [file pone.0229743.s001.doc]
