# Supplementary material for: High-mobility group box (TOX) antibody a useful tool for the identification of B and T cell subpopulations
Source: PLoS One. 2020 Feb 27;15(2):e0229743. doi: 10.1371/journal.pone.0229743 (PMC7046285; doi:10.1371/journal.pone.0229743)
Supplement: Attachment — Submitted filename: 2. Response to Reviewers.doc [file pone.0229743.s002.doc]

**Editor-in-Chief** of PlosOne

January 24^th^, 2020

## Dear Dr Joerg Heber,

Please find enclosed our revised manuscript entitled “**High-mobility group box (TOX) antibody a useful tool for the identification of B and T cell subpopulation”**.

In this new version, we have included several modifications, following the academic editor and reviewer suggestions, and we feel that these have improved our manuscript. All comments have been addressed in this revised manuscript and our responses to the editor’s and reviewer’s comments are attached. We would be grateful to have this revised manuscript reconsidered for publication in PlosOne as a regular article.

Please do not hesitate to contact me if you require any further information.

Yours sincerely

Dr Giovanna Roncador

Head of the Monoclonal Antibodies Unit,
Spanish Nacional Cancer Research Centre (CNIO)

**Reply to the comment from academic editor**

Additionally, particular emphasis must be paid to the quality of the Western blot data and it's discussion within the text. For example, Fig.2A shows only a single band for TOX, why? Is it that HEK293 cells do not post-translationally alter the protein as suggested? I also note that reference 3 cited in the manuscript never ran TOX from their 293 cells on a Western, so this does not help clarify the observed anomaly.

To address this point we have repeated the Western blot with 10µg of a cell extract of each TOX family protein (TOX, TOX2, TOX3, and TOX4) overexpressed in HEK293T cells. Using increasing concentrations of the lysate from HEK293T cells expressing recombinant TOX

(2.5µg, 5µg and 10µg) we demonstrate the presence of the second lower molecular weight form of TOX (weaker intensity) when the protein concentration is increased (5µg and 10µg).

We have included a Western blot with 10µg of the TOX HEK293T lysate in the manuscript.

Please also note the journal's relatively new policy on cropped images, whereby the original, unmodified, images for all westerns and gels must now be submitted in the supplementary data.

As requested by the editor, we have added the original and unmodified blotting images into the supplementary data. We have also added cell line authentication reports in the supporting information.

Finally, the conclusions drawn from Fig.3 are invalid. You cannot compare TOX expression between cell types whose loading controls are so vastly different. For example, to suggest RAJI express TOX while AKATA does not is simply unjustified. Indeed, loading controls are meant to be the same to show that direct comparisons can be made. I would ask the authors to repeat these Westerns to achieve the same loading levels before drawing any conclusions

As requested by the reviewer, we have repeated the Western blotting with similar levels of sample loading across the cell line panel. We have changed the Western blotting image in Figure 3 and added the uncropped original images in supporting information.

**Reply to the comment from reviewer**

1. The authors reported expression of TOX in BL cell line RAMOS and RAJI, but this latter data was not clearly represented in figure 3

The data in Figure 3 has been replaced with new Western blotting data to achieve more equal sample loading across the cell line panel and this now clearly illustrates the presence of TOX expression in the Ramos and Raji cell lines.

2. The study demonstrated expression pf TOX in germinal center B cells, however Burkitt lymphoma resulted TOX negative in most of the cases, although it`s a GC derived tumor. This data should be highlighted in the discussion section.

TOX expression was found in 33% of Burkitt lymphomas. This finding suggests that TOX expression could be restricted to a subset of GC-derived lymphomas. GC B cells are not a homogeneous population and this heterogeneity in the expression of GC related markers has previously been observed for other GC-related proteins such as GCET1/centerin and LMO2 (1, 2). Additional mechanisms that could explain some variability in the expression of the TOX protein might be genetic alterations including copy number variation such as amplification/deletion or translocations involving *TOX*, as observed in DLBCL (3) and primary central nervous system lymphoma (4). Further studies are required to elucidate the role played by TOX in Burkitt lymphomas.

This paragraph has been added to the main text.

1.[Montes-Moreno S](https://www.ncbi.nlm.nih.gov/pubmed/?term=Montes-Moreno S%5BAuthor%5D&cauthor=true&cauthor_uid=17898315)1, [Roncador G](https://www.ncbi.nlm.nih.gov/pubmed/?term=Roncador G%5BAuthor%5D&cauthor=true&cauthor_uid=17898315), [Maestre L](https://www.ncbi.nlm.nih.gov/pubmed/?term=Maestre L%5BAuthor%5D&cauthor=true&cauthor_uid=17898315), [Martínez N](https://www.ncbi.nlm.nih.gov/pubmed/?term=Martínez N%5BAuthor%5D&cauthor=true&cauthor_uid=17898315), [Sanchez-Verde L](https://www.ncbi.nlm.nih.gov/pubmed/?term=Sanchez-Verde L%5BAuthor%5D&cauthor=true&cauthor_uid=17898315), [Camacho FI](https://www.ncbi.nlm.nih.gov/pubmed/?term=Camacho FI%5BAuthor%5D&cauthor=true&cauthor_uid=17898315), [Cannata J](https://www.ncbi.nlm.nih.gov/pubmed/?term=Cannata J%5BAuthor%5D&cauthor=true&cauthor_uid=17898315), [Martinez-Torrecuadrada JL](https://www.ncbi.nlm.nih.gov/pubmed/?term=Martinez-Torrecuadrada JL%5BAuthor%5D&cauthor=true&cauthor_uid=17898315), [Shen Y](https://www.ncbi.nlm.nih.gov/pubmed/?term=Shen Y%5BAuthor%5D&cauthor=true&cauthor_uid=17898315), [Chan WC](https://www.ncbi.nlm.nih.gov/pubmed/?term=Chan WC%5BAuthor%5D&cauthor=true&cauthor_uid=17898315), [Piris MA](https://www.ncbi.nlm.nih.gov/pubmed/?term=Piris MA%5BAuthor%5D&cauthor=true&cauthor_uid=17898315).

# Gcet1 (centerin), a highly restricted marker for a subset of germinal center-derived lymphomas.

[Blood.](https://www.ncbi.nlm.nih.gov/pubmed/17898315) 2008 Jan 1;111(1):351-8.

2.Natkunam Y, Zhao S, Mason DY, et al.

(Natkunam, 2007 #146).

Blood. 2007;109(4):1636–1642.

3.Schmitz R, Wright GW, Huang DW, Johnson CA, Phelan JD, Wang JQ, et al.

Genetics and Pathogenesis of Diffuse Large B-Cell Lymphoma.

N Engl J Med. 2018;378:1396-407.

4.Cheng J, Tu P, Shi QL, Zhou HB, Zhou ZY, Zhao YC, et al. [Primary diffuse large B-cell lymphoma of central nervous system belongs to activated B-cell-like subgroup: a study of 47 cases]. Zhonghua Bing Li Xue Za Zhi. 2008;37:384-9. PMID: 19031717.

3. 65% of FL turned out to be TOX+, the authors should report the percentage of positive cases in each grade (1/2, 3A, 3B)

As suggested by the reviewer we have reported the percentage of positive cases in each grade of FL. Overall TOX expression was detected in 65% of FL and showed reduced frequency of expression in low grade 1 lymphomas (grade 1, 43%; grade 2, 73%; grade 3A, 73%; grade 3B, 67%).

These data have been added to the main text.

**We also have amended our manuscript to meet all the Journal requirements**

- We have included the full name of the animal ethics committee that approved our study in the supporting information and in the submission form.
- We have included additional information about the patient samples.
- Our GEP data was extracted from previous publications from our group (1-2)

The data from PMID 22110251 can be found in GEO GSE32233 while the data from PMID 23429603 are in process of being deposited in GEO repository.

1.Arribas AJ, Gomez-Abad C, Sanchez-Beato M, Martinez N, Dilisio L, Casado F, et al. Splenic marginal zone lymphoma: comprehensive analysis of gene expression and miRNA profiling. Mod Pathol. 2013;26:889-901. PMID: 23429603.

2.Arribas AJ, Campos-Martin Y, Gomez-Abad C, Algara P, Sanchez-Beato M, Rodriguez-Pinilla MS, et al. Nodal marginal zone lymphoma: gene expression and miRNA profiling identify diagnostic markers and potential therapeutic targets. Blood. 2012;119:e9-e21. PMID: 22110251.

- All funders had no role in study design, data collection and analysis, decision to publish, or preparation of the manuscript.
